# A low-calorie diet raises β-aminoisobutyric acid in relation to glucose regulation and leptin independent of exercise in women with obesity

**DOI:** 10.3389/fphys.2023.1210567

**Published:** 2023-06-09

**Authors:** Habiba Faiz, Steven K. Malin

**Affiliations:** ^1^ Department of Kinesiology and Health, Rutgers University, New Brunswick, NJ, United States; ^2^ University of Virginia, Charlottesville, VA, United States; ^3^ Division of Endocrinology, Metabolism and Nutrition, Rutgers University, New Brunswick, NJ, United States; ^4^ New Jersey Institute for Food, Nutrition and Health, Rutgers University, New Brunswick, NJ, United States; ^5^ Institute of Translational Medicine and Science, Rutgers University, New Brunswick, NJ, United States

**Keywords:** insulin sensitivity, pancreatic function, myokine, caloric restriction, interval exercise

## Abstract

**Introduction:** β-aminoisobutyric acid (BAIBA) is a suggested cytokine secreted from skeletal muscles that regulates insulin sensitivity, pancreatic function, and fat oxidation. However, no studies to date have examined if a low-calorie diet (LCD) or LCD + with interval exercise (LCD + INT) differentially raises BAIBA. The purpose was to examine if LCD or LCD + INT raises circulating BAIBA in relation to cardiometabolic health.

**Methods:** For this**,** twenty-three women with obesity were randomized to either 2-weeks of LCD (*n* = 12, 48.4 ± 2.5 y, 37.84 ± 1.5 kg/m^2^; ∼1200 kcal/day) or LCD + INT (*n* = 11, 47.6 ± 4.3 y, 37.9 ± 2.3 kg/m^2^; ∼60 min/d of INT alternating 3 min of 90% and 50% HRpeak), with matched energy availability. Fasting BAIBA and adipokines along with glucose, insulin, C-peptide, and FFA after every 30 min up to 120 min were obtained during a 75 g OGTT to estimate total area under the curve (tAUC), insulin sensitivity (SI_IS_), pancreatic function [disposition index (DI)], and hepatic insulin clearance (HIC). Fuel use (indirect calorimetry) was tested at 0, 60, and 120 min of the OGTT along with fitness (VO_2_peak) and body composition (BodPod).

**Results:** Both treatments lowered body weight (*p* < 0.001) and leptin (*p* < 0.001) but raised BAIBA (*p =* 0.007) and insulin sensitivity (*p* = 0.02). LCD + INT increased VO_2_peak (*p* = 0.02) and REE tAUC_120min_ (*p =* 0.02) while LCD and LCD + INT decreased carbohydrate oxidation (CHO_ox_) tAUC_120min_ (*p* < 0.001). Increased BAIBA associated with reduced weight (*r* = −0.67, *p* < 0.001), leptin (*r* = −0.66, *p* = 0.001), CHO_ox_ tAUC_120min_ (*r* = −0.44, *p* = 0.03) and DI_muscle120min_ (*r* = −0.45, *p* = 0.03), but elevated HIC_120min_ (*r* = 0.47, *p* = 0.02).

**Discussion:** Concluding, LCD and LCD + INT increased BAIBA in relation to reduced body weight and pancreatic function in women with obesity. This suggests energy deficit is a key factor regulating circulating BAIBA.

## 1 Introduction

Nearly 66% of people in the U.S. have excess weight that places them at high risk for morbidity and mortality, and women generally have more body fat than men (Hales et al., 2017). Excess adiposity is problematic for cardiometabolic health as it raises pro-inflammatory cytokines that impair insulin sensitivity (Chouchani and Kajimura, 2019). Subsequently, many people with obesity are in an energy surplus due to either a high calorie diet and/or perform low levels of physical activity (e.g., <150 mins/wk) (The Physical Activity Guidelines Advisory Committee, 2008), thereby reducing skeletal muscle glucose uptake and fat oxidation (Felber et al., 1987). A mechanism by which energy surplus and/or physical inactivity could impact skeletal muscle relates to declines in myokine secretion. Indeed, people with obesity have low myokine levels that correlate with insulin resistance, glucose intolerance and cardiometabolic disease risk (Gadde et al., 2018). Thus, identification of treatments that effectively improve skeletal muscle myokine levels could be important for chronic disease risk reduction.

Caloric restriction is a primary lifestyle factor that we and others show reduces adiposity, low-density lipoprotein (LDL), blood pressure, and inflammation as well as enhances glucose tolerance and insulin sensitivity (Gilbertson et al., 2019; Napoleão et al., 2021). Exercise also reduces body fat, which in turn, relates to enhanced adipose tissue health (Pedisic et al., 2020) as reflected by higher adiponectin and lower leptin (Heiston et al., 2020a), as well as insulin-stimulated glucose uptake, fat oxidation and pancreatic β-cell function (Stanford and Goodyear, 2018). More specifically, interval exercise (INT) has been shown to promote greater fat loss than continuous exercise (Viana et al., 2019). Further, INT may be more effective than traditional aerobic exercise in older adults to improve VO_2_peak as well as levels of PGC-1α and quality of life (Earnest, 2008). Although the exact mechanism by which a low calorie diet (LCD) or exercise impacts cardiometabolic health remains unclear, one potential factor could relate to myokine production (Chow et al., 2022). β-aminoisobutyric acid (BAIBA) is a non-protein amino acid reported to have numerous health benefits including insulin sensitivity, pancreatic function, and fat oxidation. Recent work demonstrates that a low-fat or low-carbohydrate diet intervention enhanced urinary BAIBA in relation to improved insulin sensitivity among people with overweight and obesity (Haufe et al., 2017). Further, acute aerobic exercise performed continuously for 1 h at 40% of their peak power output enhanced circulating BAIBA in recreationally active people (Stautemas et al., 2019), although others reported no such acute exercise effect (Morales et al., 2017). Nonetheless, 16 weeks of traditional aerobic exercise training was reported to raise BAIBA in normal-weight individuals as compared to those with obesity (Short et al., 2019). Together, while the literature suggests diet or exercise have abilities to raise BAIBA, no studies have examined if a LCD with interval exercise (LCD + INT) compared with LCD matched on energy availability raise BAIBA differently in people with obesity. Given we recently demonstrated that when energy availability is matched in women, a LCD compared with LCD + INT induces similar benefits on whole-body insulin sensitivity, fat oxidation, adiposopathy and metabolic syndrome risk factors (Gilbertson et al., 2019), it would be reasonable to expect BAIBA to increase comparably. Therefore, we tested the hypothesis that BAIBA levels would increase similarly following LCD + INT and LCD in women with obesity. We also sought to examine if changes in BAIBA correlated with improved insulin sensitivity, substrate oxidation, β-cell function, adipokines and hepatic insulin clearance.

## 2 Methods

### 2.1 Participants

Twenty-three women with obesity were randomized to LCD (*n* = 12; 48.4 ± 2.5 y, 37.8 ± 1.5 kg/m^2^) or LCD + INT (*n* = 11; 47.6 ± 4.3 y, 37.9 ± 2.3 kg/m^2^). Some cardiometabolic data, including those from the oral glucose tolerance test (OGTT), were previously presented (Francois et al., 2018), but are reported here for ease of interpretation. Participant recruitment was done with advertisements through local newspapers, social media, and flyers. Individuals were included if non-smokers, sedentary (<60 min of exercise/week), free of chronic diseases (e.g., T2D, liver disease, cardiac dysfunction, etc.) but with obesity (body mass index (BMI) of 30–50 kg/m^2^) and not taking any medications known to impact glycemia (e.g., biguanides, GLP-1 agonists, etc.). All participants underwent a physical examination that included resting EKG (12-lead electrocardiogram), medical history, and urine as well as blood biochemistry to ensure eligibility. Menses status was not controlled but it was documented in LCD (*n* = 5; post-menopause, *n* = 6; irregular periods, and *n* = 1; pre-menopause) and LCD + INT (*n* = 7; post-menopausal, *n* = 3; pre menopause, *n* = 1; not reported). This study was conducted according to the guidelines laid down in the Declaration of Helsinki and all procedures involving human participants were approved by the Institutional Review Board (HSR: #18316). Participants provided oral as well as written informed consent.

### 2.2 Aerobic fitness and body composition

A continuous incremental test on a cycle ergometer with indirect calorimetry (CareFusion, Vmax CART, Yorba Linda, CA, United States) was utilized to determine peak oxygen consumption (VO_2_peak). Heart rate peak (HRpeak) was also obtained from the VO_2_peak test to prescribe submaximal exercise. Individuals maintained a cadence >60 rpm and the test concluded once RER > 1.1 and volitional exhaustion was reached. A digital scale was used to measure weight, without shoes and minimal clothing. Height was determined using a stadiometer, and BMI was subsequently calculated as weight divided by height^2^. Fat mass and fat-free mass (FFM) were measured using air displacement plethysmography (BodPod, Concord, CA, United States) while waist circumference (WC) was measured 2 cm above the umbilicus using a tape measure three times and averaged.

### 2.3 Metabolic control

Participants were instructed to consume their routine diet and record it 3 days prior to pre-intervention testing. Emphasis was placed to consume 250 g of carbohydrate 24 h prior to pre-intervention testing. The participants were also instructed to refrain from consuming alcohol, caffeine, any medications and strenuous physical activity 24 h prior to the study visits ^(^(Francois et al., 2018)^)^. The last exercise bout of training was performed 24 h prior to the OGTT.

### 2.4 Oral glucose tolerance test (OGTT)

After an overnight fast of 10–12 h, participants arrived at Clinical Research Unit between approximately 06:00-08:00. Indirect calorimetry with canopy (Vmax Encore, CareFusion, Yorba Linda, CA, United States) was used to determine resting energy expenditure (REE) and substrate oxidation after rest for 20 min in a supine position. Breath samples were collected for a total of 15 min and the last 5 min of the data were averaged for analysis. An intravenous catheter was then placed in the antecubital vein for blood collection before and during the 120 min 75 g OGTT. Fasting high molecular weight (HWM) adiponectin, total adiponectin and leptin were used to assess adipose derived inflammation. Adiposopathy, i.e., “sick fat,” was defined as the ratio of total and HMW adiponectin to leptin based on prior work (Gilbertson et al., 2019). Leptin was adjusted for fat mass as well by calculating leptin/FM ratio to assess adipose function independent of body mass. Fasting BAIBA was also analyzed. Circulating glucose, insulin, free fatty acids (FFA), and C-peptides were measured at 0 min as well as additionally every 30 mins up to 120 mins as previously described (Francois et al., 2018). Peripheral, predominantly skeletal muscle, insulin sensitivity was calculated by SI_IS_ while hepatic insulin resistance was estimated via the Homeostatic Model Assessment for Insulin Resistance (HOMA-IR) by using fasting glucose multiplied by insulin divided by 405 as previously described (Bastard et al., 2009; Heiston et al., 2020b). Adipose tissue insulin resistance (Adipose-IR) was calculated by multiplying fasting FFA by fasting insulin (Malin et al., 2016). Glucose tolerance was assessed by calculating the total area under the curve (tAUC). Glucose-stimulated insulin secretion (GSIS) was calculated as C-peptide tAUC divided by glucose tAUC during the OGTT. The early (0–30 min) and total phase (0–120 min) disposition index was used to calculate β-cell function relative to skeletal muscle as tAUC of GSIS*SIIS. β-cell function adjusted for hepatic and adipose insulin resistance was also calculated as tAUC of GSIS divided by HOMR-IR or Adipose-IR, respectively. Hepatic insulin clearance (HIC) was calculated as tAUC of C-peptide divided by tAUC of insulin during the OGTT. Fuel selection was also performed at 0, 60 and 120 min of the OGTT test to characterize post-prandial metabolism.

### 2.5 Cardiometabolic risk

Metabolic syndrome (MetS) risk was defined using the National Cholesterol Education Program Adult Treatment Panel (NCEP) (ATP) III criteria. MetS was considered present if at least 3 out of 5 criteria were met: fasting plasma glucose (FPG) (≥100 mg/dL), fasting high-density lipoproteins (HDL-c) (women: <50 mg/dL), blood pressure measurement (>130/85 mmHg), triglyceride (TG) levels (≥150 mg/dL), and waist circumference (women: ≥80 cm) (Huang, 2009). As such, ATP III criteria were summed to determine number of MetS risk factors and MetS prevalence. MetS z-scores were also calculated to characterize disease severity. Female-specific *Z-*scores were calculated as: Z-score = [(50-HDL)/12.90] + [(TG-150)/55.80] + [(FPG-100)/6.72] + [(WC-88)/12.9] + [(MAP-100)/17.74].

### 2.6 Low calorie diet (LCD)

LCD was targeted at about 1200 kcal/day for 13 days. This LCD was based on the pre-operative recommended diets for adults with obesity who underwent bariatric surgery as well as aligns with LCD suggestions for weight loss and maintenance (Kim, 2021). Food logs for 3 days prior to pre-intervention testing were recorded. Meal replacement shakes were then provided to the participants for breakfast and lunch (EnsureⓇ Abbott Laboratories, United States, 8 fl. oz; providing 160 kcal, 16 g protein, 2 g fat, 19 g carbohydrate). The shake containers were collected back from the participants to assess compliance. Two 100 kcal snacks during the day were provided and participants were instructed to consume a sensible dinner and not exceed 600 kcal (e.g., lean meat with vegetables). Reference guides were also provided to the participants which displayed the serving sizes of food and beverages. 13-day food records were maintained and averaged to assess compliance along with post-intervention caloric intake. Caloric intake and macronutrient analysis was assessed using The Food Nutrition Processor Analysis Software (version 11.1; ESHA Research, Salem, OR) (Heiston et al., 2019). Absolute energy deficit was calculated by subtracting pre-intervention from post-intervention calories.

### 2.7 Low calorie diet + interval exercise (LCD + INT)

Twelve exercise sessions were conducted over 13 days for those randomized to LCD + INT along with similar LCD recommendations as that of LCD group. Exercise consisted of 60 min/d on a cycle ergometry with alternating 3 min intervals at 90% HRpeak followed by 50% HRpeak. HR was monitored constantly during exercise to assess the intensity (Polar Electro, Inc. Woodbury, NY). On day 1 and 2, participants performed exercise for 30 and 45 mins at the desired intensity for acclimation purpose. Energy expenditure per session was estimated at about 350 kcal. In order to match the calorie deficit between LCD and LCD + INT, a mixed-meal shake, (Ensure^®^Abbott Laboratories, Lake Forest, IL, United States, 8 fL. oz; providing 350 kcal, 13 g protein, 11 g fat, 50 g carbohydrate), was provided after each session.

### 2.8 Biochemical analyses

Blood lipid levels were measured using enzymatic colorimetric assays via our University Medical Laboratory. Plasma glucose was analyzed by a glucose oxidase assay (YSI Instruments 2700, Yellow Springs, OH). All remaining samples collected from blood were centrifuged for 10 mins at 15000 × g at 4°C and plasma was stored at −80°C for later analysis until later batched-analyzed in duplicate to minimize variance within conditions. Insulin, C-peptide, and FFA vacutainers contained aprotinin. Insulin and C-peptide were measured using an ELISA (Millipore, Billerica, MA). Plasma FFAs were determined by a colorimetric assay (Wako Chemicals, Richmond, VA). Total adiponectin, HMW adiponectin and leptin were assessed using an enzyme-linked immunosorbent assay (EMD Millipore, Billerica, MA, United States). BAIBA was measured by LCMS as previously described (Wilkins et al., 2019). Briefly, 20 ul of plasma sample was spiked with an internal standard solution consisting of isotopically labeled amino acid. The supernatant was immediately derivatized with 6-aminoquinolyl-N-hydroxysuccinimidyl carbamate according to Waters’ AccQ-Tag kit. A 10-point calibration standard curve underwent a similar derivatization procedure after the addition of internal standards. Both derivatized standards and the sample were analyzed on a Thermo Quantum Ultra triple quadrupole mass spectrometer coupled with a Waters Acquity liquid chromatography system. Data acquisition was done using selected reaction monitoring (SRM) via positive electrospray condition. Concentration of the analyte was calculated against its respective calibration curve per protocol at the Mayo Clinic Metabolomics Core Laboratory (Lanza et al., 2010; Wilkins et al., 2019). For example, BAIBA (SRM 274 > 171) and valine (SRM 288 > 171) metabolites were compared against the internal standards d_6_-gamma-Aminobutyric acid (SRM 280 > 171) and ^13^C_5_-Valine (SRM 293 > 171).

### 2.9 Statistical analyses

Data were analyzed using IBM SPSS Statistics (Version 28.0.0.0 (190), IBM Corp). Normality was assessed and data were log transformed when appropriate. Independent, two-tailed t-tests were utilized to measure any differences in baseline variables and absolute energy deficit. A 2-way repeated measure analysis of variance (ANOVA) was used to examine differences between LCD and LCD + INT group. Pearson and Spearman rank order correlation analyses were also performed to examine correlations when appropriate. Effect sizes were calculated for interaction (g x t) effects using eta squared (η^2^) to assess physiologic relevance, which were interpreted as small = 0.01, medium = 0.06, and large = 0.14. Data are presented as mean ± SEM. Significance was accepted at *p* ≤ 0.05.

## 3 Results

### 3.1 Cardiometabolic risk and caloric intake

Both LCD and LCD + INT decreased weight (*p* < 0.001), fat mass (*p* < 0.001), and fat free mass (*p* = 0.003*;* Table 1)*.* As expected, only LCD + INT increased VO_2_peak in an (interaction effect *p* = 0.02*,* η^2^ = 0.18*;* Table 1). Indeed, exercise adherence was about 98% and participants averaged approximately 74% and 89% HRpeak for low and high intensity exercise. There was no significant difference in the HRpeak after both interventions (*p* = 0.71*;* Table 1). Both treatments reduced total cholesterol (*p* < 0.001), triglycerides (*p* = 0.007), and LDL (*p* < 0.001*;* Table 1). HDL levels decreased in both treatments (*p* = 0.02) but more after LCD than LCD + INT (interaction effect; *p* = 0.004*, η* ^2^ = 0.38; Table 1). MetS z-score (*p* < 0.001) as well as ATP III criteria (*p* = 0.01) also decreased similarly after both treatments (Table 1). This is consistent with lower caloric intake after both treatments (*p* = 0.001; Table 1), which was not different between LCD and LCD + INT (−741.3 ± 206.2 vs. −405 ± 194.1kcal; *p* = 0.24). Moreover, carbohydrate (*p* = 0.04), fat (*p* < 0.001), and protein intake (*p* = 0.01) were all decreased comparably after both treatments (Table 1).

**TABLE 1:** Participant demographics.

	LCD		LCD + INT		*p*-value G	*p*-value T	*p*-value G x T
	PRE	POST	PRE	POST			
*Participant Characteristics*							
N (F)	12	—	11	—	—		—
Age (yrs)	48.4 ± 2.5	—	47.6 ± 4.3	—	—		—
*Body Composition*							
Weight (kg)^	102 ± 5.0	99.5 ± 4.9	104.9 ± 6.9	103.1 ± 6.9	0.91	<0.001	0.10
BMI (kg/m^2^)	37.8 ± 1.5	36.9 ± 1.5	37.9 ± 2.3	37.4 ± 2.3	0.78	<0.001	0.10
FFM (kg)	51.8 ± 1.9	51.0 ± 1.9	53.8 ± 2.7	52.9 ± 2.7	0.58	0.003	0.89
Body Fat (%)	50.0 ± 0.9	49.9 ± 0.8	47.7 ± 1.7	47.9 ± 1.7	0.29	0.94	0.44
*Aerobic Fitness*							
VO_2_peak (mL/kg/min)	18.1 ± 1.0	17.5 ± 1.1	16.5 ± 0.8	17.6 ± 0.4	0.59	0.56	0.05
*Cardiometabolic Disease Risk*							
ATPIII Score	2.17 ± 0.3	2.08 ± 0.2	2.89 ± 0.2	2.00 ± 0.2	0.30	0.01	0.03
WC (cm)	105 ± 3.0	104 ± 3.0	104.8 ± 4.7	105.0 ± 4.6	<0.001	0.70	0.53
SBP (mmHg)^	125 ± 2.6	119 ± 3.2	121.8 ± 5.1	118.6 ± 4.5	0.66	0.05	0.53
DBP (mmHg)	71.6 ± 6.8	68.3 ± 2.1	66.8 ± 6.19	66.4 ± 3.6	0.40	0.46	0.58
TG (mg/dL)	101 ± 15	81.0 ± 9.3	110 ± 12.6	81.0 ± 10.1	0.86	0.007	0.60
HDL-c (mg/dL) ^	50.7 ± 2.2	43.6 ± 1.9	49.9 ± 9.2	49.1 ± 4.6	0.003	0.02	0.004
MetS Z-Score (a.u.)	−5.8 ± 0.5	−1.1 ± 0.7	−5.74 ± 0.6	−1.88 ± 0.4	0.64	<0.001	0.20
*Caloric Intake*							
Calories (kcal)	2096 ± 202	1355 ± 31	2014 ± 179	1609 ± 98	0.58	0.001	0.13
Proteins (g)	83.3 ± 8.1	61.8 ± 3.0	78.2 ± 8.92	71.86 ± 3.7	0.73	0.01	0.17
Carbohydrates (g)	236 ± 26	175 ± 6.7	238.7 ± 29	217.6 ± 13	0.30	0.04	0.31
Fats (g)	780 ± 69	400 ± 18	775 ± 84.7	416 ± 27.5	0.93	<0.001	0.82
*Amino Acid*							
Valine (µM)	204 ± 10.2	206 ± 8.7	197 ± 10.3	198.7 ± 7.5	0.56	0.69	0.92

Data are presented as mean ± SEM. A two-way repeated measure ANOVA test was performed to find out the effect of interventions. VO_2_peak, aerobic capacity relative to mean body weight (kg) and fat free mass (FFM). ATP III, National Cholesterol Education Program Adult Treatment Panel (ATP) III criteria. WC, waist circumference. SBP, systolic blood pressure. DBP, diastolic blood pressure. FPG, fasting plasma glucose. FFA, free fatty acid. TG, triglycerides. HDL-c, high-density lipoprotein. ^Non-normally distributed data are presented in raw version for ease of interpretation. Significance accepted at *p* ≤ 0.05.

### 3.2 Insulin sensitivity, pancreatic function and fuel use

Fasting as well as tAUC_120min_ for glucose were not significantly affected by either intervention (Table 2). However, insulin tAUC_120min_ (*p* = 0.008; Table 2 ) as well as fasting and 120 min C-peptides during the OGTT (*p* = 0.03) decreased following LCD and LCD + INT. In contrast, early-phase FFA tAUC_30min_ (*p* = 0.02) as well as total phase FFA tAUC_120min_ increased following LCD and LCD + INT (*p* = 0.001, Table 2), while LCD + INT maintained FFA at 120 min as compared to increases with LCD (interaction effect*; p* = 0.01, *η* ^2^ = 0.29). Muscle insulin sensitivity (SI_IS_; *p* = 0.02*;* Table 3)*,* as well as HIC tAUC_120min_ increased after both treatments as well (*p* = 0.02; Table 3). Neither treatment impacted DI_muscle_ or DI_liver_ as well as total phase DI_adipose_ after LCD and LCD + INT (Table 3). Further, LCD + INT increased resting energy expenditure (REE) (kcal/kg/d) tAUC_120min_ (interaction effect; *p* = 0.02, *η* ^2^ = 0.21; Table 4) and maintained 120 min CHO_ox_ compared with LCD (interaction effect; *p* = 0.03, *η* ^2^ = 0.23; Table 4).

**TABLE 2:** Effect of LCD and LCD + INT on insulin, glucose, C-peptides and FFA.

	LCD		LCD + INT		*p*-value G	*p*-value T	*p*-value G x T
	PRE	POST	PRE	POST			
*Insulin*							
Fasting (uU/mL)	15.3 ± 2.0	14.4 ± 3.2	13.2 ± 1.5	12.2 ± 4.0	0.49	0.61	0.99
120min (uU/mL) ^	83.2 ± 15.0	74.2 ± 16	125.3 ± 21	80.2 ± 0.1	0.11	0.007	0.11
tAUC_30min_ (uU/mL)	2254 ± 313	1619 ± 224	1753 ± 241	2232 ± 280	0.10	0.68	0.76
tAUC_120min_ (uU/mL) ^	10783 ± 1376	9264 ± 1251	16526 ± 2986	13548 ± 2559	0.30	0.008	0.35
*Glucose*							
Fasting (mg/dL)^	97.0 ± 1.5	94.2 ± 2.5	96.9 ± 2.4	92.9 ± 2.1	0.48	0.09	0.95
120 min (mg/dL)	113.0 ± 6.3	115.3 ± 9	122.8 ± 7.0	126 ± 8.3	<0.001	0.65	0.93
tAUC_30min_ (mg/dL)	3493 ± 109	3426 ± 98	3571 ± 158	3249 ± 290	0.81	0.15	0.34
tAUC_120min_ (mg/dL)	14670 ± 754	14274 ± 880	15905 ± 900	14971 ± 667	0.38	0.08	0.46
*C-Peptides*							
Fasting (ng/mL)	2.27 ± 0.2	1.94 ± 0.2	2.16 ± 0.2	2.09 ± 2.0	0.95	0.06	0.22
120min (ng/mL)	9.0 ± 0.8	8.79 ± 0.2	12.74 ± 1.2	10.2 ± 1.2	0.06	0.03	0.07
tAUC_30min_ (ng/mL)	152 ± 12.8	149 ± 15	158.5 ± 9.8	161 ± 12	0.56	0.95	0.79
tAUC_120min_ (ng/mL) ^	967 ± 71.6	933 ± 73	1187 ± 98	1061 ± 69	0.60	0.05	0.81
*FFA*							
Fasting (mEq/L)	0.5 ± 0.03	0.6 ± 0.05	0.6 ± 0.05	0.6 ± 0.05	0.21	0.17	0.31
120min (mEq/L)	0.06 ± 0.007	0.04 ± 0.007	0.06 ± 0.01	0.08 ± 0.01	0.20	0.92	0.02
tAUC_30min_ (mEq/L)	12.67 ± 0.9	16.3 ± 1.8	14.6 ± 1.5	16.3 ± 1.4	0.58	0.02	0.41
tAUC_120min_ (mEq/L) ^	25.5 ± 2.2	33.6 ± 3.8	28.7 ± 4.0	36.0 ± 3.2	0.15	0.001	0.86
*HIC tAUC*_*30min*_	0.09 ± 0.0	0.09 ± 0.0	0.08 ± 0.0	0.07 ± 0.03	0.07	0.76	0.31
*HIC tAUC*_*120min*_	0.09 ± 0.007	0.1 ± 0.007	0.08 ± 0.008	0.09 ± 0.008	0.16	0.02	0.58

Data are presented as mean ± SEM. A two-way repeated measures ANOVA was performed to find out the effect of interventions. FFA , free fatty acids. HIC, hepatic insulin clearance; Insulin tAUC/C-peptide tAUC. tAUC, total area under the curve. ^Non-normally distributed data are presented in raw version for ease of interpretation. Significance accepted at *p* ≤ 0.05.

**TABLE 3:** Effect of LCD and LCD + INT on insulin sensitivity and secretion.

	LCD			LCD + INT		*p*-value G	*p*-value T	*p*-value G x T
	PRE		POST	PRE	POST			
*Insulin Sensitivity*								
SI_IS_	0.19 ± 0.002		0.2 ± 0.002	0.19 ± 0.002	0.19 ± 0.003	0.16	0.02	0.54
HOMA-IR	3.70 ± 0.5		3.47 ± 0.8	3.83 ± 0.8	3.60 ± 0.9	0.90	0.61	0.98
Adipose-IR	7.78 ± 1.3		7.82 ± 1.5	7.33 ± 0.6	7.31 ± 0.7	0.78	0.98	0.97
Insulin Secretion								
GSIS30min	0.04 ± 0.003		0.04 ± 0.004	0.05 ± 0.003	0.05 ± 0.005	0.34	0.60	0.53
GSIS120min	0.07 ± 0.005		0.07 ± 0.005	0.07 ± 0.003	0.11 ± 0.04	0.23	0.32	0.33
DImuscle30min	0.008 ± 0.00		0.008 ± 0.007	0.009 ± 0.00	0.009 ± 0.00	0.34	0.75	0.68
DImuscle120min	0.01 ± 0.0009		0.01 ± 0.001	0.01 ± 0.0004	0.01 ± 0.0003	0.95	0.75	0.53
DIliver30min	0.013 ± 0.001		0.018 ± 0.003	0.014 ± 0.002	0.041 ± 0.001	0.68	0.13	0.31
DIliver120min ^	0.02 ± 0.001		0.03 ± 0.008	0.02 ± 0.003	0.02 ± 0.002	0.60	0.16	0.55
DIadipose30min	0.0057 ± 0.0		0.0055 ± 0.0	0.0056 ± 0.0	0.0053 ± 0.0	0.94	0.62	0.82
DIadipose120min	0.009 ± 0.001		0.0080 ± 0.00	0.0085 ± 0.001	0.008 ± 0.001	0.94	0.34	0.70

Data are presented as mean ± SEM. A two-way repeated measures ANOVA was performed to find out the effect of interventions. SI_IS_, simple index for insulin sensitivity reflects muscle. HOMA-IR, homeostatic model assessment of insulin resistance reflects liver. Adipose-IR, adipose insulin resistance. GSIS, glucose stimulated insulin secretion. DI, disposition index. ^Non-normally distributed data are presented in raw version for ease of interpretation. Significance accepted at *p* ≤ 0.05.

**TABLE 4:** Effect of LCD and LCD + INT on fuel use.

	LCD		LCD + INT		*p*-value G	*p*-value T	*p*-value G x T
	PRE	POST	PRE	POST			
*CHO*_*ox*_							
Fasting (mg/kg/min)	1.03 ± 0.1	0.67 ± 0.1	0.7 ± 0.1	0.57 ± 0.1	0.07	0.04	0.21
120min (mg/kg/min)	1.80 ± 0.1	1.12 ± 0.2	1.2 ± 0.1	1.22 ± 0.2	0.16	0.02	0.03
tAUC_120min_ (mg/kg/min)	187 ± 13	119 ± 18	143 ± 19	94 ± 15.4	0.09	<0.001	0.30
*F*_*ox*_							
Fasting (mg/kg/min)	0.5 ± 0.04	0.6 ± 0.05	0.5 ± 0.05	0.6 ± 0.05	0.12	0.007	0.92
120min (mg/kg/min) ^	0.3 ± 0.03	0.2 ± 0.02	0.3 ± 0.05	0.1 ± 0.04	0.56	<0.001	0.22
tAUC_120min_ (mg/kg/min)	121 ± 7.4	76.7 ± 8.3	103 ± 12	90.9 ± 8.3	0.83	0.01	0.13
*REE*							
Fasting (kcal/kg/day)	11.2 ± 0.8	11.7 ± 0.01	11.0 ± 0.8	12.8 ± 0.01	0.95	0.07	0.49
120min (kcal/kg/day)	13.5 ± 0.5	12.3 ± 0.4	11.5 ± 0.6	12.4 ± 0.6	0.12	0.004	0.38
tAUC_120min_ (kcal/kg/day)	1577 ± 64	1480 ± 41	1367 ± 84	1488 ± 63	0.21	0.79	0.02

Data are presented as mean ± SEM. A two-way repeated measures ANOVA was performed to find out the effect of interventions. CHO_ox_, carbohydrate oxidation. F_ox_, fat oxidation. REE, resting energy expenditure. tAUC, total area under the curve. ^Non-normally distributed data are presented in raw version for ease of interpretation. Significance accepted at *p* ≤ 0.05.

### 3.3 BAIBA and adipokines

**FIGURE 1:**
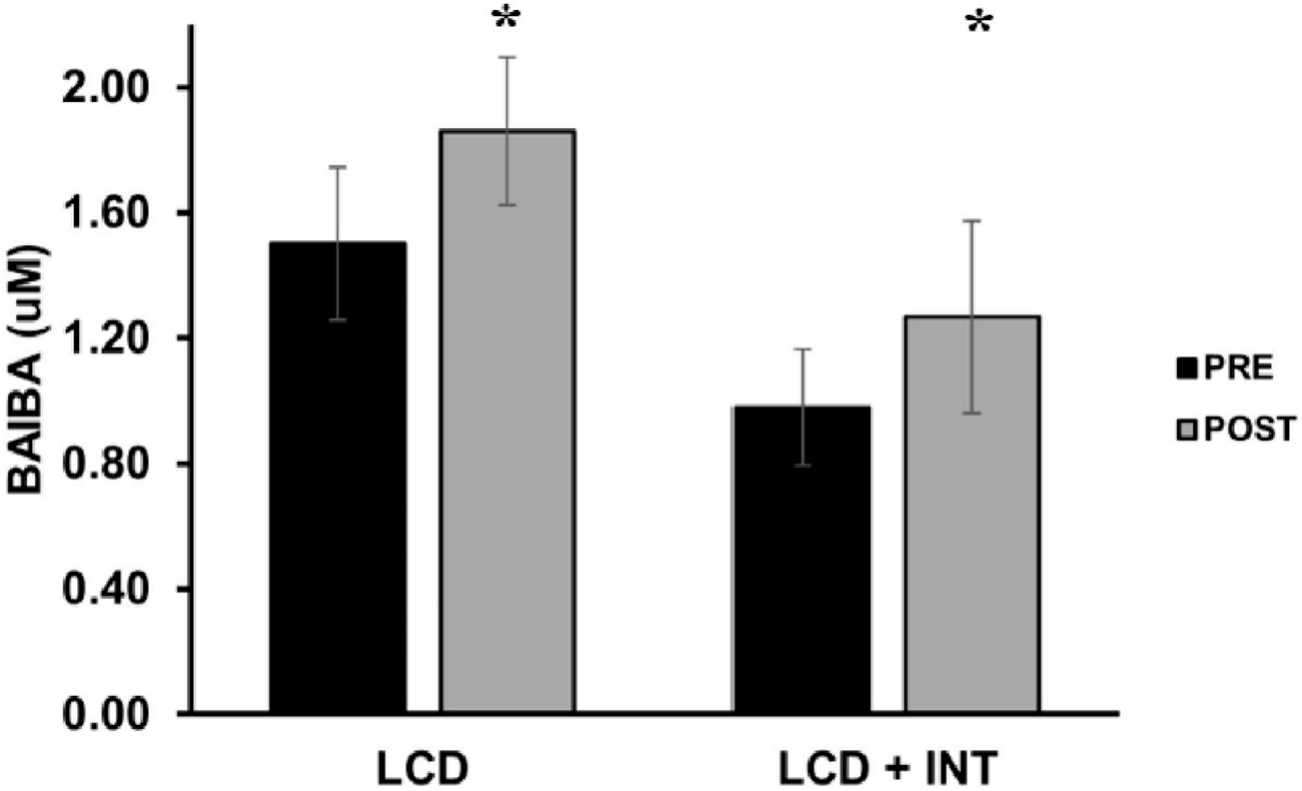
Effect of LCD and LCD + INT on BAIBA. BAIBA was non-normally distributed, and data are presented in raw version for ease of interpretation. Data are presented as mean ± SEM. *Significant time effect (*p* = 0.007).

While both LCD and LCD + INT raised BAIBA (*p* = 0.007), there was no difference between treatments (*p* = 0.50; *η* ^2^ = 0.02; Figure 1). Plasma leptin levels were reduced following each treatment (*p* < 0.001; Table 5), and this decline in leptin remained after scaling data to fat mass after both interventions (*p* = 0.003)*.* There was no effect on total adiponectin, but both interventions increased HMW adiponectin (*p* = 0.05; Table 5) as well as HMW adiponectin/leptin ratio (*p* = 0.002) and total adiponectin/leptin ratio (*p* = 0.02; Table 5)*.*

**TABLE 5:** Effect of LCD and LCD + INT on adipokines.

	LCD		LCD + INT		*p*-value G	*p*-value T	*p*-value G x T
	PRE	POST	PRE	POST			
*Adipokines*							
Total Adiponectin (ng/mL) ^	11759 ± 1879	11377 ± 1869	13670 ± 2993	13341 ± 2263	0.61	0.42	0.62
HMW Adiponectin (ng/mL) ^	4122 ± 751	4087 ± 652	4157 ± 1036	5838 ± 1383	0.99	0.05	0.28
Leptin (ng/mL)	81.5 ± 9.6	54.0 ± 6.9	77.2 ± 5.9	61.3 ± 8.0	0.89	<0.001	0.26
Total Adiponectin/Leptin	161 ± 30.2	257 ± 54.6	133 ± 26.7	155 ± 28.2	0.23	0.02	0.15
HMW Adiponectin/Leptin	49.8 ± 10.8	88.3 ± 18.6	51.4 ± 10.6	73.5 ± 16.3	0.73	0.002	0.33

Data are presented as mean ± SEM. A two-way repeated measures ANOVA was performed to find out the effect of interventions. ^Non-normally distributed data presented in raw version for easy interpretation. HMW, high molecular weight. FM, fat mass. Total adiponectin/leptin and HMW adiponectin/leptin ratios represent adiposopathy. ^Non-normally distributed data are presented in raw version for ease of interpretation. Significance accepted at *p* ≤ 0.05.

### 3.4 Correlations

**FIGURE 2:**
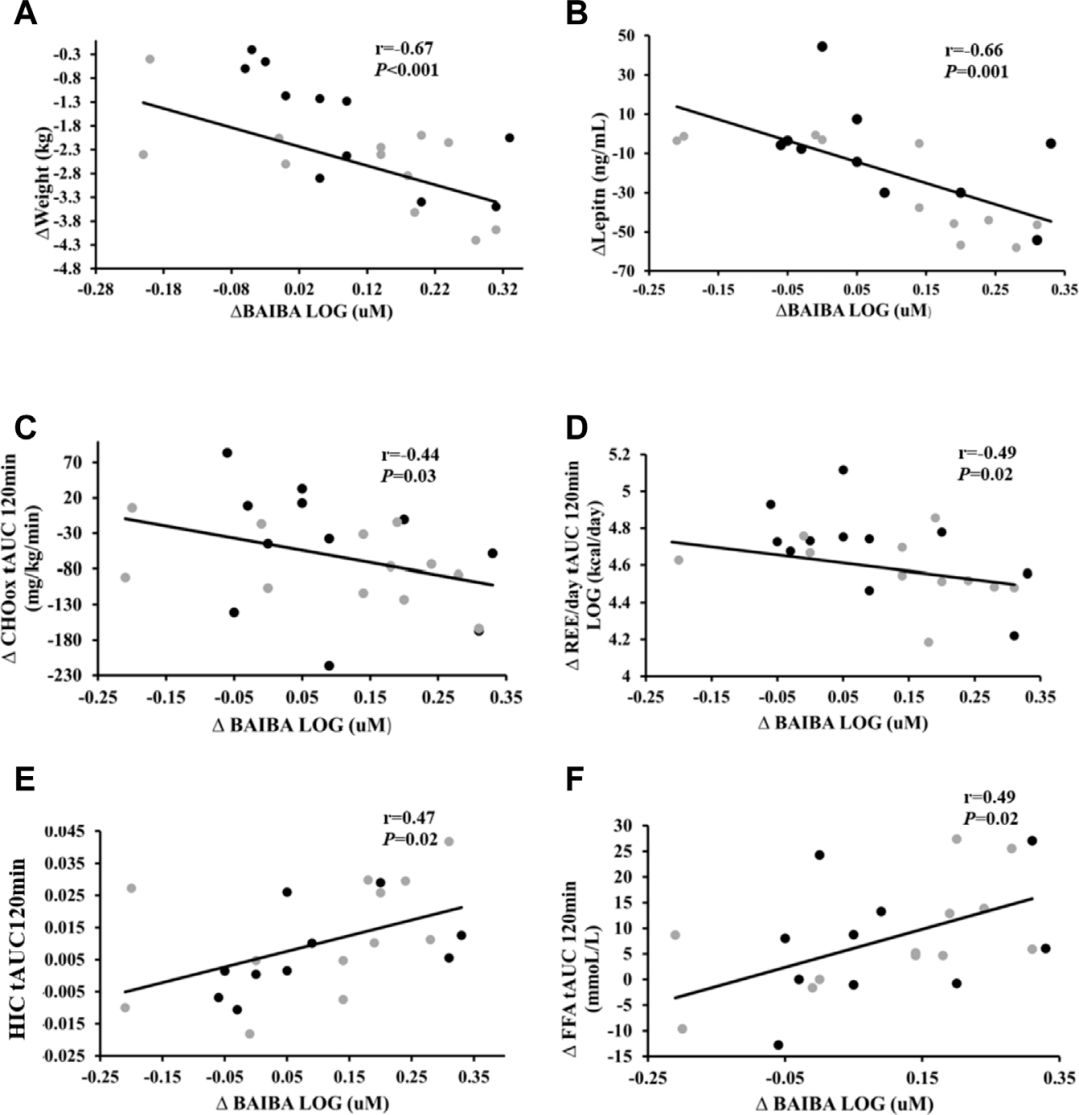
Correlations of change in BAIBA and cardiometabolic health. Correlation of change (Δ) in BAIBA to **(A)** Δ weight, **(B)** Δ leptin levels, **(C)** Δ Carbohydrate oxidation (CHOox) tAUC_120min_, **(D)** Δ Resting energy expenditure (REE/day) tAUC_120min_ **(E)** Δ Hepatic insulin clearance (HIC) tAUC_120min_, and **(F)** Δ Free fatty acids (FFA) tAUC_l20min_. Grey circles- LCD, black circles- LCD + INT. Significance accepted at *p* < 0.05.

Increased BAIBA correlated with weight loss (*r* = −0.67, *p* < 0.001), reduced leptin levels (*r* = −0.66, *p* = 0.001), lower total phase DI_muscle_ (*r* = −0.45, *p* = 0.03), REE tAUC_120min_ (kcal/day) (*r* = −0.49, *p* = 0.02), and CHO_ox_ tAUC_120min_ (*r* = −0.44, *p* = 0.03) but higher F_ox_ tAUC_120min_ (*r* = 0.45, *p* = 0.04: Figure 2). Higher BAIBA also correlated with higher FFA tAUC_30min_ (*r* = 0.45, *p* = 0.03) but lower C-peptide tAUC_30min_ (*r* = −0.43, *p* = 0.04) and insulin tAUC_30min_ (*r* = −0.50, *p* = 0.01; Figure 2). Moreover, BAIBA correlated with increased VO_2_peak (*r* = 0.45, *p* = 0.02), FFA tAUC_120min_ (*r* = 0.49, *p* = 0.02), and HIC tAUC_120min_ (*r* = 0.47, *p* = 0.02). Interestingly, increases leptin correlated with rises in VO_2_peak (*r* = 0.60, *p* = 0.006) and REE tAUC_120min_ (kcal/day) (*r* = 0.47, *p* = 0.03).

## 4 Discussion

The main finding of this work demonstrates that a 2-week intervention of LCD or LCD + INT increases plasma BAIBA levels in women with obesity. This might be clinically relevant since lower BAIBA levels are reported in people with obesity (Roberts et al., 2014; Short et al., 2019), particularly total body fat and sub-cutaneous fat tissue (Rietman et al., 2016). Our work showing similar increases in BAIBA following both LCD and LCD + INT matched on energy availability is consistent with work reporting that caloric restriction via low-fat or low-carbohydrate treatment on BAIBA in overweight and obese individuals enhanced BAIBA levels (Haufe et al., 2017). In fact, even a low-gluten diet supporting weight loss increased urinary BAIBA levels in healthy Danish adults (Hansen et al., 2018). A knowledge gap from this work on caloric deficit is whether exercise could potentiate the effect of diet on BAIBA. In fact, 20 weeks (3 d/wk) of aerobic exercise in sedentary but healthy individuals, increased circulating BAIBA (Roberts et al., 2014). This aligns with others showing that 16 weeks of aerobic exercise raises BAIBA levels by 29% in people with normal-weight as compared to those with obesity (Short et al., 2019). In contrast, while some report no change in plasma BAIBA in humans after cycling at 70% VO_2_peak ^(^(Morales et al., 2017)^)^, others observed elevated circulating BAIBA following 60 min of acute exercise (Stautemas et al., 2019). It is hard to reconcile this later acute exercise work, but it is worth noting that BAIBA release after exercise might only be enhanced in muscles exercised (Barlow et al., 2020) and consideration of different exercise modes may be important for enhancing BAIBA. Nevertheless, similar to work in children showing that both diet and physical activity raise BAIBA levels (Miccheli et al., 2015), our findings extend the literature by highlighting not only does a LCD diet in women with obesity raise BAIBA, but adding interval exercise does not further enhance this rise in BAIBA. Thus, our collective work shows for the first time that energy deficit achieved by diet, with or without exercise, increases BAIBA in women with obesity.

There are several other reasons why LCD and LCD + INT could have raised BAIBA. BAIBA is produced from the catabolism of either valine (S-BAIBA) by ABAT enzyme or thymine (R-BAIBA) by AGXT2 enzyme. Valine catabolism takes place in the muscle primarily while thymine degradation is occurring in the liver (Stautemas et al., 2019). That means protein oxidation of skeletal muscle could be important for influencing BAIBA. If individuals lose FFM during lifestyle therapy, then it would be reasonable to anticipate higher valine catabolism could contribute to higher circulating BAIBA (Steinhauser et al., 2018). While we did not directly measure protein oxidation or whole-body protein turnover in the current study, there was no impact on plasma valine concentrations despite both LCD and LCD + INT inducing similar weight/fat and FFM loss. Interestingly, we observed direct correlations with weight loss and BAIBA. This suggests change in body mass is linked to elevations in BAIBA and additional work is needed to elucidate how BAIBA levels change with lifestyle therapy. Another possibility is related to fat mass. Both treatments reduced leptin and increased HMW adiponectin levels, suggesting that our treatments improved adiposopathy, or adipose function. Indeed, participants with obesity have high levels of leptin in part through leptin resistance. Weight loss is suggested to lower plasma leptin/raise leptin sensitivity and contribute to weight regulation (Izquierdo et al., 2019). In our study, higher BAIBA was related to lower leptin levels. This finding is consistent with work in mice showing that BAIBA might act through a leptin dependent mechanism and implies that BAIBA may either directly or indirectly impact adipose function (Begriche et al., 2008). Given that leptin scaled to fat mass remained lower following both treatments, our findings imply that the higher BAIBA may directly link to lower leptin via a muscle-adipose crosstalk mechanism.

Elevated BAIBA could influence oxidative capacity and both LCD as well as LCD + INT are thought to influence fuel use. It has been suggested that BAIBA contributes to low adiposity by browning of the white adipose tissue, elevations in metabolic rate (Begriche et al., 2008) and enhanced fat oxidation (Roberts et al., 2014). BAIBA induced body fat loss has been attributed to enhanced aerobic energy expenditure in mice, which corresponds to enhanced FFA oxidation in the liver and skeletal muscle (Tanianskii et al., 2019). Moreover, *in vitro* testing shows that BAIBA enhances hepatic β-oxidation, due to in part, increased β-oxidation gene (PPAR-α) expression (Roberts et al., 2014). In the current study, rises in VO_2_peak were associated with increased BAIBA. This finding, while consistent with the connection of oxidative metabolism and BAIBA, is somewhat surprising given the LCD + INT treatment did not raise BAIBA more than LCD. This suggests that gains in fitness may play secondary roles in influencing BAIBA and additional work is needed to confirm given no exercise only condition was studied herein. Nevertheless, insulin sensitive muscle is capable of taking up glucose to either store and/or oxidize glucose as fuel. In line with gains in insulin sensitivity observed in this present work, BAIBA correlated with reduced insulin tAUC. It is important to acknowledge though that higher BAIBA levels correlated with lower early phase C-peptide tAUC. This implies that BAIBA may relate to pancreatic function, which contributed to lower insulin levels. Interestingly, recent work suggests that BAIBA decreased acute insulin release from INS-1832/3 cells when challenged with submaximal hyperglycemia and lower BAIBA levels were correlated with insulin secretory function in humans (Barlow et al., 2020). Herein, we observed that rises in BAIBA correlated with reductions in GSIS adjusted for both skeletal muscle and adipose. Since muscle insulin sensitivity increased and Adipose-IR did not change, the decrease in disposition index in relation to BAIBA could imply BAIBA acts to assist in the coordination of tissues to dispose-off glucose into muscle while enabling adipose lipolysis to support FFA delivery for energy metabolism (Shi et al., 2016; Chen et al., 2021). Interestingly, we do report that BAIBA correlated with less CHO_ox_ and increased F_ox_ tAUC during the OGTT. Hence our findings identify BAIBA as a potential factor coordinating bodily storage of glucose during the post-prandial state while concurrently using fat for energy.

The present study has some limitations worth noting. The modest sample size limits the ability to generalize findings, and inclusion of women only do not allow extrapolation of these findings to men. Associations also do not equal causation and additional investigation into the mechanism by which BAIBA acts is needed. We measured total BAIBA and cannot discern independent effects of R-, compared with S-, BAIBA enantiomer. R-BAIBA is primarily produced in the liver and kidney whereas S-BAIBA is produced primarily in the muscle and also, in the liver during acute exercise ^(^(Stautemas et al., 2019)^)^. In a study with male and female participants without a chronic illness, they did not see any association of S-BAIBA with physical performance (6-min walk tests, gait speed, grip strength, etc.,), but did observe strong correlation to BMI, fat mass and lean mass. For R-BAIBA, however, they observed it was correlated to physical performance (Lyssikatos et al., 2023). Further, exercise in this study was aerobic in nature and it would be of interest for future work to determine the impact of resistance exercise on BAIBA. Additionally, it would be important for longer-term exercise interventions to examine BAIBA in effort to confirm if rises if aerobic fitness accentuate BAIBA concentrations compared with short-term exercise (e.g., 2 wk vs. 16 wk). We used an OGTT instead of a euglycemic-hyperinsulinemic or hyperglycemic clamp to assess insulin sensitivity and insulin secretion, respectively. While the OGTT is valid and provides physiologic insight and practical relevance in assessing insulin sensitivity and pancreatic function (Bastard et al., 2007), using clamp approaches would more accurately depict insulin sensitivity and pancreatic function. Nonetheless, a strength of the study is women were randomized to treatments and attempts were made to match energy availability between groups to note if LCD + INT raised BAIBA more than LCD. With a mean difference of 0.07 between LCD and LCD + INT and SD of 0.45 at power of 0.80 and significance at 0.05, we would need 650 people per group to detect a difference. Thus, we are confident these treatments were both able to raise BAIBA.

In conclusion, our study demonstrates that LCD, with or without INT exercise, increases plasma BAIBA in women with obesity. This improvement in BAIBA correlated with weight loss, adiposopathy, energy metabolism as well as circulating insulin and pancreatic function. Together, this work highlights that energy deficit is effective at increasing BAIBA in relation to cardiometabolic health. Future work is required to determine the exact mechanism by which diet and/or exercise therapies impact BAIBA to optimize treatment that prevents/delays obesity, T2D and cardiovascular disease.

## Data Availability

The raw data supporting the conclusion of this article will be made available by the authors, without undue reservation.
